# Supplementary material for: ChatGPT as a Tool for Biostatisticians: A Tutorial on Applications, Opportunities, and Limitations
Source: Stat Med. 2025 Oct 23;44(23-24):e70263. doi: 10.1002/sim.70263 (PMC12548020; doi:10.1002/sim.70263)
Supplement: Supplementary file 1 — Data S1. Supporting Information. [file SIM-44-0-s001.zip › Supplementary Materials Additional.zip!/3.6_Simulation_Study/3.6_Simulation_Chat3.pdf]

Coverage prob3 - Coverage Probability Simulation

Take the role of an expert biostatistician for this chat. The goal is to perform a simulation study to assess the coverage probability of a confidence interval with nominal coverage 95%. The setting is a case control study design, which is a retrospective observational study that compares individuals with a disease (cases) to those without it (controls) to identify potential risk factors or exposures associated with the disease.

We aim at the coverage probability of an appropriate effect size estimate in a parametric model. Propose a suitable exact confidence interval and investigate its empirical coverage under the null hypothesis of no effect. Use 5000 Monte Carlo runs and the sample sizes must vary between 20 and 40. Provide results for ten different settings with different sample sizes and marginal probabilities. Be consize in your answer and do not provide unnecessary descriptions.

To evaluate the empirical coverage probability of a **95% confidence interval (CI)** for an effect estimate in a **case-control study**, we use **logistic regression** with the **odds ratio (OR)** as the effect size.

Study Design

- Binary outcome $Y \in \{0, 1\}$ (case vs. control)
- Binary exposure $X \in \{0, 1\}$
- Under H_0: No association, i.e., $\text{OR} = 1$ or $\log(\text{OR}) = 0$
- CI based on **Wald method** for $\log(\text{OR})$:
  $\hat{\beta} \pm 1.96 \cdot \text{SE}(\hat{\beta})$
- Exact CI evaluated empirically via **Monte Carlo simulation**

Simulation Setup

- 5000 repetitions per scenario
- Ten combinations of:
  - Total sample size $N \in [20, 40]$
  - Case proportion $\pi_Y \in \{0.3, 0.5, 0.7\}$
  - Exposure probability $\pi_X = 0.5$

Pseudocode Summary

1. For each setting:
   - Generate N binary outcomes with fixed case/control ratio.
   - Generate exposure $X \sim \text{Bernoulli}(\pi_X)$, independently of Y.

- Fit logistic regression $Y \sim X$, extract $\hat{\beta}, \mathrm{SE}$
- Compute Wald CI and check if it contains 0

2. Estimate empirical coverage as proportion of CIs covering 0.

Python Implementation (Core)

```python
import numpy as np
import pandas as pd
import statsmodels.api as sm
from tqdm import tqdm
import ace_tools as tools

np.random.seed(42)

def simulate_coverage(n, pi_y, pi_x, n_sim=5000):
    coverages = []
    n_cases = int(n * pi_y)
    n_controls = n - n_cases
    for _ in range(n_sim):
        y = np.array([1]*n_cases + [0]*n_controls)
        x = np.random.binomial(1, pi_x, size=n)
        try:
            model = sm.Logit(y, sm.add_constant(x)).fit(disp=0)
            beta = model.params[1]
            se = model.bse[1]
            ci_lower = beta - 1.96 * se
            ci_upper = beta + 1.96 * se
            coverages.append(ci_lower <= 0 <= ci_upper)
        except:
            coverages.append(False)
    return np.mean(coverages)

# Settings
settings = [
    (20, 0.3), (20, 0.5), (20, 0.7),
    (25, 0.3), (25, 0.5), (25, 0.7),
    (30, 0.4), (35, 0.5), (40, 0.4), (40, 0.6)
]

results = []
for n, pi_y in tqdm(settings):
    coverage = simulate_coverage(n, pi_y, pi_x=0.5)
    results.append({'n': n, 'pi_y': pi_y, 'coverage': coverage})

df_results = pd.DataFrame(results)
tools.display_dataframe_to_user(name="Coverage Probability Results", dataframe=df_results)
```

Output

The resulting table provides empirical coverage probabilities for each combination of sample size and case proportion. The goal is to assess how well the Wald CI maintains nominal 95% coverage under small sample settings.
